# Rapid identification of novel antigens of *Salmonella* Enteritidis by microarray-based immunoscreening

**DOI:** 10.1007/s00604-014-1197-6

**Published:** 2014-02-14

**Authors:** Lena Danckert, Sebastian Hoppe, Frank F. Bier, Markus von Nickisch-Rosenegk

**Affiliations:** Fraunhofer IBMT, Am Mühlenberg 13., 14476 Potsdam, Germany

**Keywords:** Salmonella, Microarray, Antigen detection

## Abstract

We report on an approach to rapidly screen thousands of *Salmonella* Enteritidis proteins with the goal of identifying novel immunodominant proteins. We used a microarray-based system that warrants high throughput and easy handling. Seven immunogenic candidates were selected after screening. Comparative analyses by ELISA and microarrays manifested their immunodominant character. The large repetitive protein (SEN4030) that plays a role as a putative adhesin in initial cell surface interaction and is highly specific to *Salmonella* is considered to be the most suitable protein for a diagnostic approach. The results further demonstrate that the strategy applied herein is convenient for specifically identifying immunogenic proteins of pathogenic microorganisms. Consequently, it enables a sound assessment of promising candidates for diagnostic applications and vaccine development. Moreover, the elucidation of immunogenic proteins may assist in unveiling unknown virulence-associated factors, thus furthering the understanding of the underlying pathogenicity of *Salmonella* in general, and of *S.* Enteritidis, one of the most frequently detected serovars of this pathogen, in particular.

**Figure:**
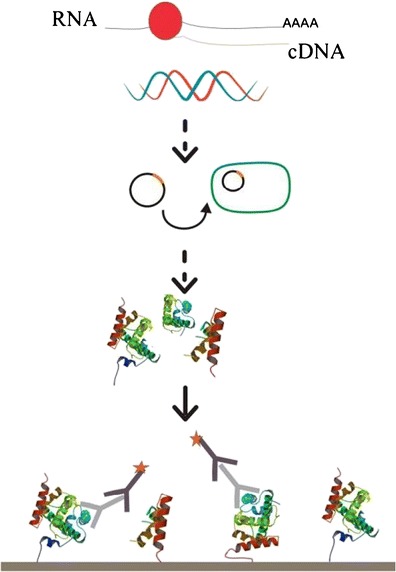
The microarray-based approach was aimed at identifying novel immunodominant proteins of *S.* Enteritidis. Seven antigens were revealed by screening a cDNA expression library. SEN4030, a large repetitive protein specific for salmonella, is considered an optimal candidate for future applications.

The microarray-based approach was aimed at identifying novel immunodominant proteins of *S.* Enteritidis. Seven antigens were revealed by screening a cDNA expression library. SEN4030, a large repetitive protein specific for salmonella, is considered an optimal candidate for future applications.

## Introduction

*Salmonella* are Gram-negative, facultative anaerobe, motile and rod-shaped bacteria comprised of three species, *S. enterica*, *S. bongori* and *S. subterranean*. However, many different serovars have been described for *S. enterica* including the major contributors to salmonella infections in humans, S. Enteritidis, S. Typhi, S. Typhimurium, S. Paratyphi, and S. Choleraesuis. While S. Typhi and S. Paratyphi cause typhoid fever, S. Enteritidis and S. Typhimurium lead to gastrointestinal infections termed salmonellosis [1]. In the European Union alone, approximately 100.000 human cases of salmonellosis are reported annually, with *S.* Enteritidis and *S.* Typhimurium the most frequently detected serotypes (EFSA, 2013). These non-typhoidal salmonella (NTS) cause a localized infection manifesting as nausea, vomiting, abdominal cramps, diarrhea and fever. The infection dose is approximately 10^5^ bacteria and the disease is mainly self-limiting with mild symptoms [2]. However, in immunocompromised people and young children the severity of the disease may be more pronounced including typhoid-like infections potentially leading to systemic infections and sepsis [3]. While several in vivo animal infection models have been used to study the pathogenicity of S. Typhimurium [4–6], *S.* Enteritidis has been insufficiently studied. Additionally, evidence suggests that S. Enteritidis requires genes missing in S. Typhimurium [7].

The detection of enteric pathogens relies primarily on standard cultivation techniques. The bacteria are cultured from food or fecal samples and detection comprises pre-enrichment, enrichment, identification of the pathogen and confirmation as mandatory steps, which usually take several days [8]. Although standard cultivation tests are dependable and well-established, the demand for more rapid diagnostic tools is high. Especially during the containment of epidemics, isolation of patients in hospitals, and monitoring of contaminations in food-processing plants time is critical. Therefore, immunoassay-based tests, e.g. ELISA or lateral flow tests deserve consideration. Whereas ELISA is a laboratory-intensive method that takes roughly 4–6 h, lateral flow test strips are designed with easy handling and read-out in mind. In fact, immunochromogenic strips (ICS) based on lateral flow have been successfully introduced in the developing countries to detect *Treponema pallidum*, the cause for the sexually transmitted infection syphilis, among others [9]. Commonly used ELISA systems are based on a sandwich assay format using Anti-O-H antibodies [10], which detect both the O-antigen (polysaccharide) and the H-antigen (flagella) of salmonella. However, in order to improve sensitivity and specificity for future tests, a deeper understanding of specific salmonella antigens is required.

In this study, we applied a method [11] based upon a cDNA expression library with subsequent immunoscreening of recombinantly expressed fusion proteins on microarrays. The expressed fusion constructs comprise an N-terminal HaloTag® [12] and the C-terminal salmonella proteins. The former is a Dehalogenase derivative that provides a covalent, irreversible and highly specific binding to its corresponding ligand [13]. The high binding affinity enables a direct application of the cell lysate rendering time consuming purification steps obsolete [14]. Consequently, seven novel antigens of S. Enterititdis were identified.

## Experimental

### Bacterial strains and growth media

*S*. Enteritidis strain 125109 (phage-type 4) was used for immunoscreening and the *E. coli* electrocompetent cell lines Acella™ (www.edgebio.com/, Gaithersburg, MD) and KRX single-step competent cells (www.promega.de*/*) were used for cloning. *S*. Enteritidis was grown in Nutrient Broth 2 (www.sigmaaldrich.com/) and *E. coli* in Lysogeny broth (LB) with addition of ampicillin (100 μg mL^−1^).

### cDNA library construction

All steps describing RNA isolation, polyadenylation and normalization of RNA, cDNA synthesis, ligation-independent cloning and transformation via electroporation have already been reported elsewhere [11]. After plating the transformation reactions, a total of 1536 cDNA clones including three positive controls (different KRX cells expressing FimA) and five negative controls (KRX cells expressing GapA from *K. pneumoniae* and *C. jejuni*, KRX und Acella^™^ cells without insert and LB medium) were selected via sterile tooth picks and cultivated for 16 h at 37 °C and 100 rpm in 96 DeepWell^™^ plates (www.thermofisher.com/) containing 850 μL LB-amp. The plates were centrifuged for 6 min at 2,000×*g* and the supernatant was discarded. The pellets were resuspended in 370 μL fresh LB-amp medium. Thereof 100 μL were transferred to new 96 DeepWell^™^ plates with 700 μL LB-amp and incubated for 3.5 h at 37 °C and 100 rpm. The remaining 270 μL of each sample were mixed with 30 μL of sterile-filtered DMSO and stored at −80 °C.

### Protein expression and lysis

After incubating cells for 3.5 h at 37 °C, protein expression was induced by addition of IPTG (1 mM) or rhamnose (0.1 %) and continued for 16 h at 20 °C and 100 rpm. Cells were lysed by EasyLyse™ Bacterial Protein Extraction Solution (www.epibio.com/). Briefly, plates were centrifuged for 6 min at 2,000×*g*, the supernatant discarded and the plates chilled at −20 °C for 20 min. The pellet was resuspended in 160 μL EasyLyse™ buffer consisting of 0.5 mL distilled water, 2 μL MgCl_2_ solution (1 M), 0.5 mL EasyLyse™ lysis buffer and 1 μL EasyLyse™ enzyme mix. Additionally, DNase I (8 U mL^−1^) in DNase buffer (10 mM Tris-Cl, 2.5 mM MgCl_2_, 10 mM CaCl_2_) was added to the reaction mix, reducing the viscosity of the solution.

### Microarray-based immunoscreening

The crude lysates were directly applied to HaloLink^™^ Slides (www.promega.de*/*) using the QArray2 microarray spotter (www.moleculardevices.com/). Each sample was spotted as a fourfold replicate per subarray with two identical subarrays encompassing 384 different samples per microarray. For each slide triplicates were generated. After spotting, the slides were incubated for 1 h at 65 % humidity and room temperature to allow for covalent binding of the fusion constructs to the HaloLink^™^ surface. Next, the slides were washed three times with PBST (Dulbecco’s phosphate buffered saline + 0.05 % Tween-20). Afterwards, a 2-well Proplate^™^ module (http://www.gracebio.com/) was attached to each slide to generate two independent compartments for incubation. Rabbit polyclonal IgG to *S. enterica* (BP1063P, www.acris-antikoerper.de/) was added to the top chamber with a concentration of 2 μg mL^−1^ in PBS. The bottom chamber was filled with PBS only. Incubation proceeded for 2 h at room temperature with mild rocking. After washing the slides three times with PBST, secondary antibody (Goat-polyclonal to Rabbit IgG conjugated with Chromeo^™^-546, www.abcam.com/, 5 μg mL^−1^ in PBS) was subjected to each chamber. The slides were incubated for 2 h at room temperature in the dark. After washing the slides for three times with PBST, they were rinsed with deionized water, the Proplate^™^ modules removed and the slides dried by nitrogen flow. Scanning was performed on an Axon Genepix 4200A laser scanner (www.moleculardevices.com/) with the following settings: 532 nm laser, PMT gain 400, 40 % laser power, lines to average 1, 10 μm resolution and standard green emission filter at 575 nm.

In contrast, for analyses of the identified full-length proteins, 10 × 10 arrays were constructed incorporating fivefold replicates for each sample. Sixteen identical arrays were applied per slide and analyzed independently by attaching a 16-well ProPlate^™^ module. The following antibodies were used: Rabbit polyclonal IgG Anti-*S. enterica* (ab35156, www.abcam.com/ and BP1063P, www.acris-antikoerper.de/), Rabbit polyclonal IgG Anti-BL21 *E. coli* (#322, www.micromol.com/) and two Rabbit polyclonal IgG Anti-*Klebsiella* (ab20947, www.abcam.com/ and AP00792PU-N, www.acris-antikoerper.de/). The Anti-*S. enterica* antibodies were generated by immunizing rabbits with an extract of whole cells and partially lyzed cells of *S.* Enteritidis, *S.* Typhimurium and *S.* Heidelberg.

### Data analysis

After scanning, the raw data of each slide was obtained by Axon GenePix Pro 6.1 software. For data analysis, the median fluorescence intensity (F_532_ median) of each spot corrected by the local background (B_532_) was used. In order to account for nonspecific binding of the secondary antibody, corrected fluorescence intensity, termed relative fluorescence intensity (RFI), was calculated as follows:

$$ RFI={\left({\tilde{F}}_{532}-{B}_{532}\right)}_{primary+ \sec ondary\kern0.5em  antibody}\kern-14.7em -{\left({\tilde{F}}_{532}-{B}_{532}\right)}_{\sec ondary\kern0.5em  antibody} $$

Afterwards, RFI was used to calculate a contrast value:

$$ contrast=\frac{\left( RF{I}_{sample}-R\tilde{F}{I}_n\right)}{\left( RF{I}_{sample}+R\tilde{F}{I}_n\right)} $$

with RFI_sample_ the value for each sample and RFI_n_ representing the intensity of a negative control protein.

For comparative analyses of full-length proteins the equation was slightly altered by replacing the RFI_n_ by RFI_all_ representing the median intensity of all samples within a chamber. In order to determine the proteins with potential immunogenic character a cut-off was calculated based on the limit of detection definition by IUPAC [15]:

$$ LOD=\mu +3\cdot \sigma $$

In this equation μ represents the arithmetic mean, while σ indicates standard deviation. However, when using contrast values, the above equation needs to be inserted into the contrast equation to yield the limit of detection for contrast values:

$$ LOD(contrast)=\frac{ LOD-R\tilde{F}{I}_n}{ LOD-R\tilde{F}{I}_n}=\frac{\left(R\tilde{F}{I}_n+3\cdot {\sigma}_n\right)-R\tilde{F}{I}_n}{\left(R\tilde{F}{I}_n+3\cdot {\sigma}_n\right)+R\tilde{F}{I}_n} $$

### DNA sequencing

Sequencing of isolated plasmids was done by LGC Genomics. The sequence data was evaluated by Geneious Pro 5.6.5 [16] using the BLAST [17] algorithm and the nucleotide database nr/nt.

### Synthesis of full-length proteins

Full-length genes were amplified from genomic DNA of *S*. Enteritidis. Gene specific primers were designed using Primer3 [18] within Geneious Pro 5.6.5 [16]. The GATAACGCGATCGCC sequence was added to the 5′ end of the forward primers and the CGAATTCGTTTAAAC sequence to the 5′ end of the reverse primers, respectively. The annealing temperature was calculated using the Phusion Tm calculator (https://www.neb.com/) based on the method by Breslauer [19]. PCR was performed accordingly: initial denaturation 98 °C for 3 min, 30 cycles of 98 °C for 10s, annealing temperature (calculated T_m_ + 3 °C of the lower primer) for 30 s, 72 °C for 2 min and a final extension 72 °C for 3 min. Successful amplification of full-length genes was examined by agarose gel electrophoresis (1 % agarose and 0,5 ng mL^−1^ Roti-Safe Gel Stain (Roth) in a PerfectBlue Gelsystem Mini (www.peqlab.de/) and 1× TAE running buffer). HyperLadder I (www.bioline.com/) served as a size standard. The full-length genes were purified with QIAquick PCR Purification Kit (www.qiagen.com/). Linearization of vector was performed with IF 18A F (5′ GTTTAAACGAATTCGGGCTC 3′) and IF 18A R (5′ GGCGATCGCGTTATCGCTCTG 3′). Cloning, transformation and colony pcr commenced as described in [11].

### SDS-PAGE

SDS-PAGE was carried out with full-length HaloTag™ fusion proteins. 2 μL lysate, 1 μL HaloTag® Alexa 488 ligand (10 μM) and 7 μL PBS were mixed and incubated for 30 min at room temperature in the dark. Next, 7.5 μL of this reaction were transferred to 7.5 μL SDS loading buffer composed of 3 μL 5× Protein-loading Buffer, 0.75 μL 20× Reducing agent and 3.75 μL water. The mixture was incubated for 4 min at 70 °C. Gel electrophoresis was performed with Mini-PROTEAN® TGX™ Precast Gel (any kD, 15 wells) in the Mini-PROTEAN® System (www.bio-rad.com/). BenchMark™ Fluorescent Protein Standard was used as a size reference. The gel was run for 30 min at 200 V in 1× Tris-Glycine SDS. Afterwards, fusion proteins were visualized by excitation of the fluorescent label at 473 nm using a Typhoon FLA 9000 (www3.gehealthcare.de/) imager.

### ELISA

For indirect ELISA measurements the lysate was purified. HaloTag® Protein Purification System and Magne™ HaloTag® Beads were applied according to the manufacturer’s instructions. The samples were diluted to a total protein content of 20 μg mL^−1^ in PBS and 50 μL of each sample was added to MaxiSorb® Plates (Nunc). Each sample was analyzed at least in triplicate. The ELISA plates were incubated overnight at 4 °C in a humidity chamber. After five washing steps with PBST, 200 μL 5 % non-fat dried milk in PBS was added to each well. This blocking procedure commenced for 2 h. Next, plates were washed three times with PBST and 100 μL primary antibody solution (*c* = 4 μg mL^−1^) in PBS containing 1 % non-fat dried milk were applied to each well using the respective desired antibody or PBS for controls. The plates were incubated for 2 h at room temperature and washed four times with PBST. Then, 100 μL of conjugated secondary antibody (Goat polyclonal to Rabbit IgG conjugated with Horseradish peroxidase, ab6721, www.abcam.com/, *c* = 20 ng mL^−1^) were added to each well and incubation carried on for 1 h. Finally, plates were washed once again four times with PBST and 100 μL 3,3′,5,5′-Tetramethylbenzidine (TMB, www.sigmaaldrich.com/) was added to each well for detection. After 30 min of incubation the reaction was stopped by applying 100 μL of 2 M H_2_SO_4_. The optical density was measured using the OMEGA Fluostar (www.bmglabtech.com/) at a wavelength of 450 nm.

## Results and discussion

### cDNA library construction and screening

Screening of 1536 different samples led to 192 clones being sequenced due to their fluorescence intensity. Within this group nine genes encoding proteins with immunogenic potential were revealed. In spite of that, numerous sequenced clones displayed only gene fragments ranging from 45 to 444 bp resulting in poor and ambiguous identification after BLAST analysis. This might have been caused by the excessive degradation of RNA prior to cDNA synthesis as attributed by a RNA integrity number (RIN) of 2.6. Although comparative studies have shown the RNA isolation method used to be reliable [20], it may have been a source for degradation. Still, this has been a proven method in revealing immunogenic proteins from pathogens and extracting RNA with high quality [11]. Although, cDNA synthesis and PCR amplification may have assisted in creating short fragments, the approach presented herein was specifically selected due to its ability to generate full-length [21] and high quality cDNA [22].

Nevertheless, normalization of cDNA prior to cloning was successful. Duplex-specific nuclease digests solely double-stranded DNA and has been used for normalization of cDNA prior to RNAseq [23]. This is a mandatory step to reduce the highly abundant rRNA, which encompasses more than 95 % of a total RNA extraction. As no distinct band of 3,000 bp, representing cDNA derived from 23S rRNA, is visible after gel electrophoresis, the cDNA was effectively normalized. Rather, a homogeneous smear, representing cDNA molecules of different sizes, was detected. Contrary to eukaryotic mRNA harboring a poly(A) tail, prokaryotic mRNA lacks this feature and thus cannot be directly reverse transcribed using oligo(dT) primers. However, as the applied cDNA synthesis demanded the presence of a poly(A) tail, the total RNA had to be polyadenylated prior to first-strand synthesis. After normalization, cloning commenced by introducing the inserts into linearized vector using ligation-independent-cloning (LIC) [24]. The LIC is a powerful tool in contrast to original cloning methods based upon ligase and restriction endonucleases, which often suffer from low efficiencies [25] caused mainly by short overhangs leading to nonspecific interactions and advocating religation of the vector. In contrast, LIC guarantees directional cloning at any desired site with high efficiency. Furthermore, the proteins of interest were expressed as fusion constructs harboring an N-terminal HaloTag®. This enabled covalent, irreversible binding to the microarray surface in a highly specific manner. Thus, purification was obsolete and cross-reactivity reduced to a minimum [14]. Commonly, microarray-based immunoscreenings offer high throughput of samples as Zhu, et al., 2006 [26] have shown. However, most methods incorporate time-consuming and costly purification steps prior to spotting purified target protein to nitrocellulose microarrays. The combination of high-throughput microarray-based immunoscreenings and the easy handling of the HaloTag® system warrants a fast identification of immunogenic proteins [14]. Consequently, the immunoscreenings of 1536 proteins are completed within a few hours.

Table 1 summarizes the nine protein candidates identified via microarray screening. The proteins encompass a highly conserved DNA primase that shows homology in all bacteria. Moreover, two hypothetical proteins (SEN1186 and SEN2464) were detected with no known function rendering these candidates highly attractive for further investigations. SEN2464 is additionally described as a methionine tRNA cytidine acetyltransferase providing precise recognition of the AUG codon for elongation-specific methionine tRNA [27]. For SEN4030, a large repetitive protein with 5559 amino acid residues, no function is known; however, it shows high similarity to SiiE of *S*. Typhimurium. SiiE is a giant non-fimbrial adhesin that facilitates initial interaction to the intestinal epithelium [28]. SEN4030 and the putative isomerase (SEN1504) are conserved in *Salmonella* [27]. Membrane-association is another intriguing feature as it enhances the protein’s accessibility in a diagnostic assay. This is true for the ais protein (SEN2278), the multidrug resistance protein A (SEN2659) and the membrane-bound lytic murein transglycosylase A precursor (SEN2832). The ais protein is a lipopolysaccharide core heptose(II)-phosphate phosphatase catalyzing the dephosphorylation of heptose(II) of the outer membrane lipopolysaccharide core. On top of that, it has just recently been identified as a virulence factor contributing to enteric infection [29]. Last but not least, SEN1186 is a DNA mismatch endonuclease located in the cytoplasm.

**Table 1:** List of all identified immunogenic proteins from cDNA expression library screening. The candidates are listed according to their locus tag, protein name, length and their conservation in bacteria [27]

Locus tag	Protein	Length (bp)	Size (kDa)	Conserved in (Total genera)
SEN1019	Patch repair protein	471	18	Bacteria (17)
SEN2464	Conserved hypothetical protein	2,019	74	Enterobacteriaceae (19)
SEN3053	DNA Primase	1,746	65	Gammaproteobacteria (51)
SEN1186	Conserved hypothetical protein	240	9	Enterobacteriaceae (15)
SEN1504	Putative isomerase	1,174	41	Salmonella (1)
SEN2278	Ais Protein	606	22	Enterobacteriaceae (5)
SEN2659	Multidrug resistance protein A	1,173	43	Enterobacteriaceae (18)
SEN2832	Membrane-bound lytic murein transglycosylase A precursor	1,098	40	Gammaproteobacteria (32)
SEN4030	Large repetitive protein	16,680	595	Salmonella (1)

### Analysis of immunogenic protein candidates

**Fig. 1:**
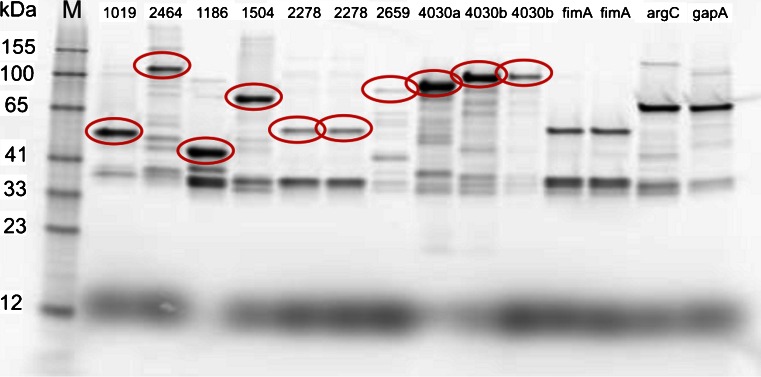
Validation of correct expression of the full-length candidates (*marked red*) and the controls via SDS-PAGE. The *E. coli* lysate was treated with HaloTag® Alexa 488 fluorescent ligand. The fusion proteins were visualized with excitation at 450 nm. As a size reference (M) BenchMark™ Fluorescent Protein Standard was added to the gel

The length of the cDNA inserts within the library ranged from 45 to 444 bp of the corresponding genes resulting in partially expressed proteins. However, after initial identification, clones expressing full-length proteins were obtained for all genes except SEN4030. SEN4030 comprises approximately 17,000 bp, so cloning of the full-length gene was not achieved. Consequently, two sequence segments, one in the central region of the gene (6,600–8,100 bp = SEN4030a) and another at the C-terminal end (13,500–15,500 bp = SEN4030b) of the gene were chosen. The 444 bp segment identified during initial immunoscreening is located within SEN4030b. Both parts contain bacterial immunoglobulin-like domains that are mainly found in bacterial surface proteins involved in pathogenicity [30]. The proteins primase and membrane-bound lytic murein transglycosylase A were excluded from further analyses due to their high homology among bacteria (>30 genera). Identity of the full-length genes was ascertained by sequencing. The expression of correct fusion constructs was determined via SDS-PAGE, see Fig. 1. All proteins were successfully expressed as fusion constructs showing the correct size including the protein of interest and the 34 kDa HaloTag™. In contrast to the other proteins, SEN2278 and SEN2659 show lower intensities in PAGE. Nevertheless, this was expected as membrane proteins tend to agglomerate more easily due to the presence of extensive hydrophobic regions. Agglomeration leads to the formation of inclusion bodies, which remain inaccessible after lysis without special treatment.

**Fig. 2:**
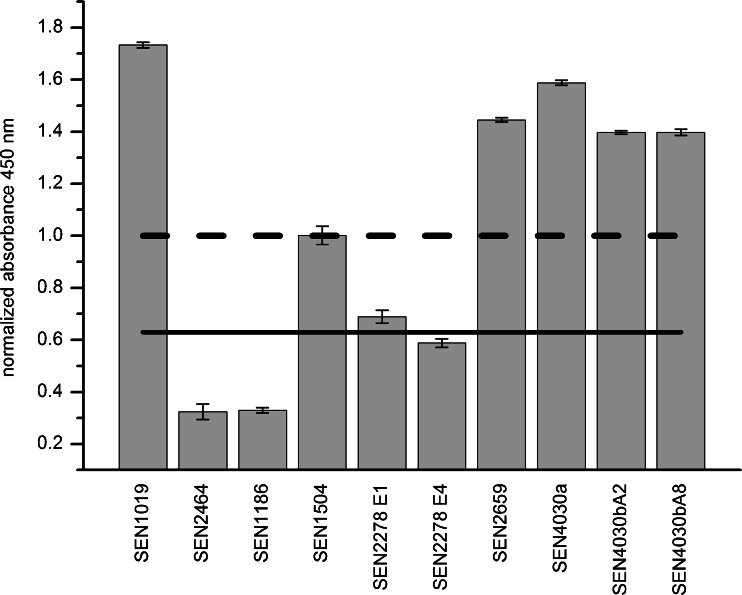
ELISA of full-length purified proteins to analyze their immunogenic character using a rabbit polyclonal IgG to *S. enterica* antibody (Acris). The extinction values are normalized on the reference immunogenic protein FimA (*dotted line*). As a negative reference, signal intensity of expression cells was used (*solid line*). The most prominent intensities are reached by the endonuclease SEN1019, the membrane-associated large repetitive protein (SEN4030a & b) and the multidrug resistance protein A (SEN2659) membrane protein

**Fig. 3:**
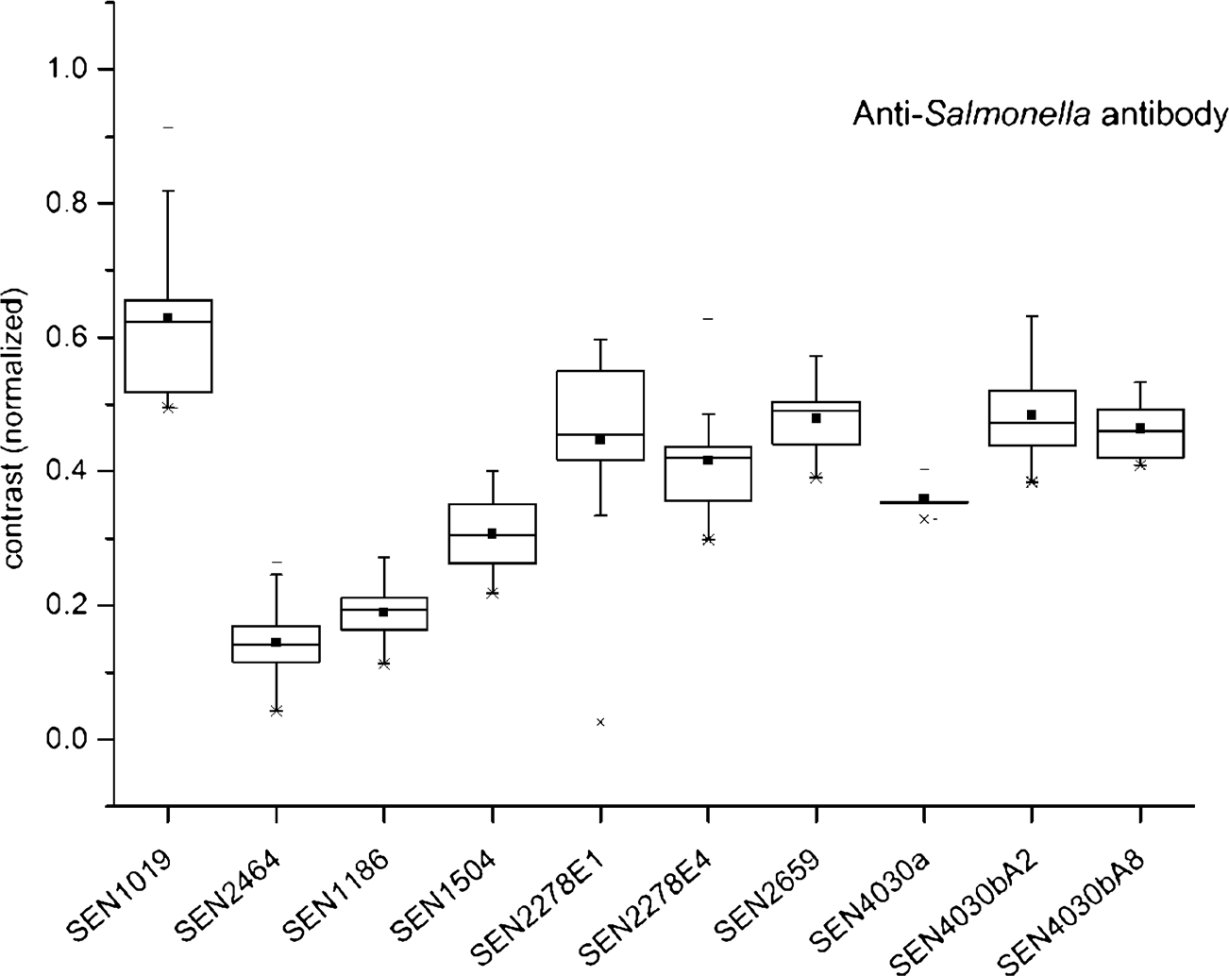
Box-Whisker Plot of the protein candidates after microarray analyses (*n* = 12). Each box encompasses 50 % of the signals, while the whiskers engulf the outliers. The black square represents the mean, whereas the median is indicated by a horizontal line. The proteins were treated with two Anti-*S. enterica* antibodies. The contrast_total samples_ ratio was normalized on the immunogenic protein FimA (Type-1 fimbrial protein, A chain)

Comparative analyses by ELISA and microarrays were performed to underline the immunodominant character of the proteins. SEN1019 scored the highest intensities with a mean of 1.7 in ELISA, followed by both parts of the large repetitive protein SEN4030 (Fig. 2). Indirect ELISA measurements require purified proteins, which are added in a defined amount. During purification the HaloTag® is cleaved off by a special endonuclease and target proteins are immobilized undirected on the well surface. On the contrary, fusion proteins are subjected directly after lysis without additional purification in microarray experiments thanks to the specific interaction of HaloTag® and its ligand on the microarray surface. Still, microarray-based immunoscreenings achieved similar results, see Fig. 3. The box-whisker-plot shows the contrast values (*n* = 12) normalized to the known immunogenic protein FimA, a type-1 fimbriae involved in bacterial attachment to epithelial cells [31]. All mean values were below 1; yet, the endonuclease SEN1019 displayed the highest mean intensity with 0.63. The investigated segments of SEN4030, SEN2659 and SEN2278 displayed intensities ranging from 0.4 to 0.5. Comparing both ELISA and microarray analyses, the normalized intensities differ in the two methods applied. Whereas SEN1019, SEN2659, and both parts of SEN4030 reached normalized intensities above 1, i.e. displaying a higher intensity than the positive reference FimA, in ELISA measurements, the intensities were below 1 in microarray analysis. Despite these differences, both methods show similar tendencies regarding protein candidates with immunodominant character. Additionally, microarray analysis combined results from two different antibodies reactive to *S. enterica*, while ELISA measurements were performed with one antibody only. Moreover, ELISA measurements represent only one fourth of the data in comparison to microarray analysis. This might have caused the observable variance in the results. Although, some uncertainty remains regarding the specificity of the polyclonal antibodies to the investigated proteins, the use of polyclonal mixtures is a prerequisite for broad initial screenings aiming at identifying novel antigens.

The full-length large repetitive protein (SEN4030) might show higher intensities during microarray analysis due to the enhancing effects of correct folding and the presence of a plethora of immunoglobulin (Ig)-like domains [28]. It is 98.6 % identical to SiiE, yet 76 amino acids are unique. SiiE is part of the *Salmonella* pathogenicity island 4 (SPI4) encoding for a type I secretion system, which secretes the adhesin SiiE. However, the protein is also partially surface-anchored [32]. Bioinformatic analysis revealed a variety of Ig-like domains and fibronectin (type III) domains. Both domains are found in surface proteins characteristic for protein-protein interactions [33]. 53 bacterial Ig-like domains are required for contact to cell surfaces. Deletion of ten or more bacterial Ig-like domains have been shown to result in a reduced infectivity [28]. The fold is stabilized by Ca^2+^ binding, which is not essential but still affects SiiE structure and function [34]. As a result of the high sequence similarity the large repetitive protein (SEN4030) of *S*. Enteritidis can be considered a putative adhesin.

## Conclusion

We have detected and identified seven novel immunogenic proteins from *S. enterica*. The method used provides fast and accessible identification of antigens within two weeks starting from cDNA library construction. Furthermore, it allows for the potential illumination of virulence-associated factors, like SEN2278 and SEN4030. These proteins influence pathogenicity in *Salmonella* and are potential candidates for *Salmonella-*specific diagnostics. Nevertheless, further analyses characterizing the epitope binding regions and comprehending the gene expression levels, especially during infection, are necessary. Furthermore, determining the affinity and specificity of generated antibodies are essential prior to diagnostic application. Still, as immunogenicity assays on microarrays and ELISA have revealed, four proteins showed prominent immunogenicity. The highest intensity was obtained for the endonuclease (SEN1019), followed by a large repetitive protein (SEN4030), the multidrug resistance protein A (SEN2659), and the ais protein (SEN2278). Membrane-association of the latter three proteins grants an ideal prerequisite for effective point-of care diagnostics. Furthermore, specificity is required for clinical applications, thus *Salmonella* conserved proteins - the large repetitive protein (SEN4030) and the putative isomerase (SEN1504) – represent optimal candidates for future endeavors. Consequently, as the large repetitive protein combines these key features it shows the highest probability to be outstandingly relevant for a diagnostic purpose.
